# Differential infection properties of three inducible prophages from an epidemic strain of *Pseudomonas aeruginosa*

**DOI:** 10.1186/1471-2180-12-216

**Published:** 2012-09-21

**Authors:** Chloe E James, Joanne L Fothergill, Hannes Kade, Amanda J Hall, Jennifer Cottell, Michael A Brockhurst, Craig Winstanley

**Affiliations:** 1 Institute of Infection and Global Health, University of Liverpool, 8 West Derby Street, Liverpool, L69 7BE, UK; 2 Department of Biology, University of York, York, YO10 5DD, UK

**Keywords:** *Pseudomonas aeruginosa*, Prophage induction, Bacteriophage infection, Cystic fibrosis, Host range, Type IV pili

## Abstract

**Background:**

*Pseudomonas aeruginosa* is the most common bacterial pathogen infecting the lungs of patients with cystic fibrosis (CF). The Liverpool Epidemic Strain (LES) is transmissible, capable of superseding other *P. aeruginosa* populations and is associated with increased morbidity. Previously, multiple inducible prophages have been found to coexist in the LES chromosome and to constitute a major component of the accessory genome not found in other sequenced *P. aerugionosa* strains. LES phages confer a competitive advantage in a rat model of chronic lung infection and may, therefore underpin LES prevalence. Here the infective properties of three LES phages were characterised.

**Results:**

This study focuses on three of the five active prophages (LESφ2, LESφ3 and LESφ4) that are members of the *Siphoviridae.* All were induced from LESB58 by norfloxacin. Lytic production of LESφ2 was considerably higher than that of LESφ3 and LESφ4. Each phage was capable of both lytic and lysogenic infection of the susceptible *P. aeruginosa* host, PAO1, producing phage-specific plaque morphologies. In the PAO1 host background, the LESφ2 prophage conferred immunity against LESφ3 infection and reduced susceptibility to LESφ4 infection. Each prophage was less stable in the PAO1 chromosome with substantially higher rates of spontaneous phage production than when residing in the native LESB58 host. We show that LES phages are capable of horizontal gene transfer by infecting *P. aeruginosa* strains from different sources and that type IV pili are required for infection by all three phages.

**Conclusions:**

Multiple inducible prophages with diverse infection properties have been maintained in the LES genome. Our data suggest that LESφ2 is more sensitive to induction into the lytic cycle or has a more efficient replicative cycle than the other LES phages.

## Background

*Pseudomonas aeruginosa* is a versatile Gram-negative bacterium, able to metabolise multiple carbon sources and exploit diverse ecological niches, *e.g.* soil, water, plants and animal hosts [1,2]. This opportunistic pathogen causes a range of human infections, including acute infections of severe wounds [3] and burns [4,5] and chronic lung infections in cystic fibrosis (CF) patients [6]. *P. aeruginosa* forms biofilms in the CF lung that are highly resistant to antibiotics and clearance by the immune system [7]. Once established, such biofilms cannot be eradicated and are associated with greatly increased morbidity and mortality [8].

Several CF-associated transmissible strains of *P. aeruginosa*, capable of between patient transmission, have been identified in the UK, Europe, Australia and North America [9]. The Liverpool Epidemic Strain (LES), a UK transmissible strain, was first isolated in 1996 at Alder Hey Children’s Hospital (AHCH), Liverpool [10]. This strain is capable of super-infection, supplanting pre-existing *P. aeruginosa* populations in the CF lung [11]. Chronic infection with LES is associated with increased morbidity and mortality compared to other *P. aeruginosa* strains [12]. The LES is highly prevalent within individual hospital CF units [13] and is the most abundant *P. aeruginosa* strain amongst CF patients in the UK [14]. It was also recently isolated from the sputa of CF patients in North America [15].

Sequencing of the earliest LES isolate, LESB58, demonstrated that the genome shares 95% similarity with the lab strain PAO1. However, its core genome is punctuated by multiple norfloxacin-inducible prophages [16]. Specifically, there are five inducible prophage genomes (LESφ2; LESφ3 LESφ4 LESφ5 and LESφ6) that are mosaic in nature. The gene organisation of LESφ2 and LESφ3 resembles that of lambdoid phages. These two phage genomes share 82.2% identity across a 13.6-kb region at their 3’ ends that makes up 32% of the phage genomes. The closest known relative to both these phages is the *Pseudomonas* phage F10 [17]. LESφ3 also contains a 7.5 kb region that shares 99.8% homology with LESφ5, which exhibits a considerable sequence similarity to the O-antigen converting phage D3 [18]. LESφ4 is a transposable Mu-like phage that closely resembles phage D3112 [19]. The LESφ6 sequence resembles a pf1-like filamentous phage [16].

Temperate phages have been shown to confer selective, beneficial traits to a range of *P. aeruginosa* hosts [20]. For example, phage D3 orchestrates O antigen conversion from O5 to O16 in PAO1, which may aid evasion of the immune system and resistance to phage superinfection [18,21]. Phage φCTX infection of PAS10 results in conversion to a toxigenic strain [22] and the filamentous phage, Pf1, has been associated with biofilm disruption and dispersal [23]. LES prophages have been suggested to contribute to the competitiveness of their bacterial host *in vivo*. LESB58 mutants, with disrupted prophage genes, exhibited 10 to 1000-fold decreased competitiveness in a rat model of chronic lung infection compared to wild type LESB58 [16]. The LES phages are induced by exposure to clinically relevant antibiotics, *e.g.* ciprofloxacin [24], and free LES phages and other tailed-phage virions have been detected in CF patient sputa [25,26].

Temperate phages are key vectors of horizontal gene transfer (HGT) [27]. Therefore, it is important to assess the ability of the LES phages to infect other bacterial hosts to which they may confer traits beneficial to life in the CF lung environment. Here we describe the infection characteristics of three of the five LES prophages LESφ2, LESφ3 and LESφ4, induced from the sequenced CF lung isolate LESB58.

## Results

### LES phage morphology

Three different *Siphoviridae* phages were induced from LESB58 cultures and visualised using electron microscopy. The phages possessed icosahedral heads (50–60 nm diameter) and long flexible tails (approximately 200 nm). Plaque assay of each phage on PAO1 resulted in the formation of small turbid plaques with different phage-specific morphologies. LESφ3 plaques were the largest (2–3 mm), with well-defined lysogen islands, whereas LESφ2 plaques were considerably smaller (0.5-1.5 mm). LESφ4 produced plaques with small, clear centres surrounded by a turbid halo. The identity of each LES phage responsible for the different plaque morphologies was confirmed using a multiplex PCR assay.

### Differential induction of LES phages from LESB58

**Figure 1:**
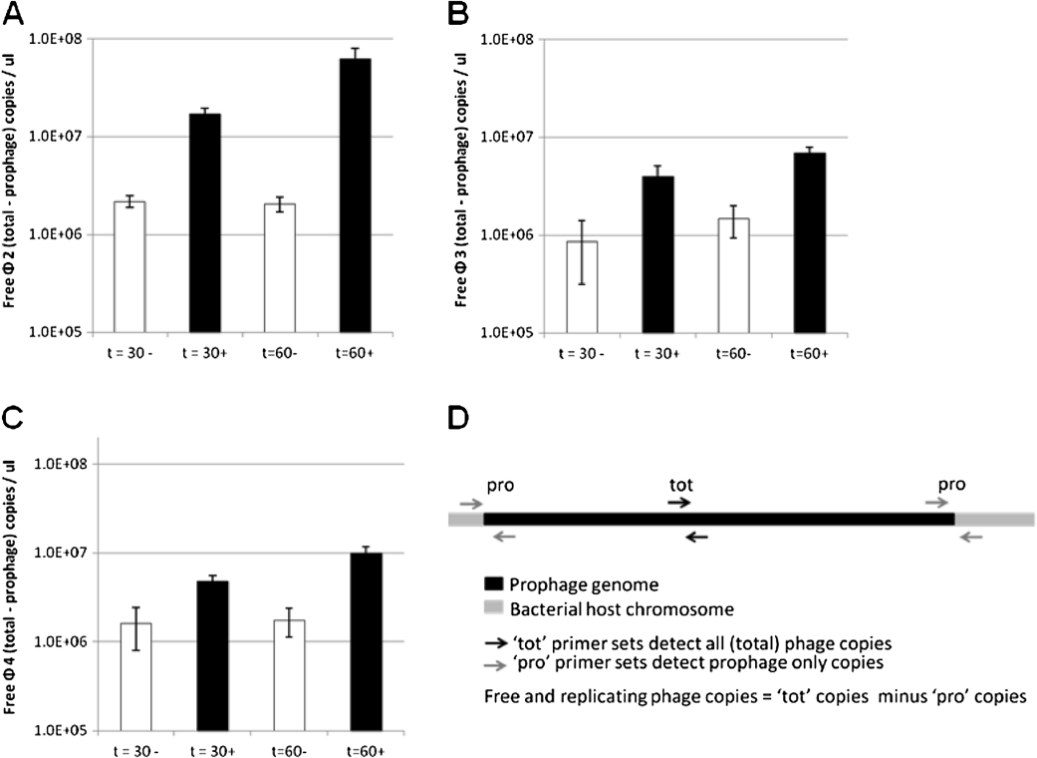
**Exposure to sub-inhibitory concentrations of norfloxacin induces the lytic cycle of three LES phages.** Mid-exponential phase LESB58 cultures (OD_600_ 0.5) were exposed to sub-inhibitory norfloxacin (50 ug ml^-1^) for 30 and 60 min before recovery for 2 h and total DNA extraction. Total phage vs prophage numbers were quantified by Q-PCR with SYBR green and specific primers. Graphs show the production levels of each phage over time; **A**: LESφ2; **B**: LESφ3; **C**: LESφ4. ■ + norfloxacin; □ – norfloxacin. **D**: Quantities of free phage were calculated by deducting prophage numbers from total phage numbers. The average free phage numbers at each time interval were plotted and Standard error is shown. Three independent experimental repeats were performed, each with 3 technical repeats.

The sensitivity of the LES phages to induction into the lytic cycle was determined and compared. Real-time quantitative (Q)-PCR was used to measure relative increases in phage DNA copy number following induction by exposure of LESB58 to norfloxacin. After exposure to norfloxacin for 60 min and recovery for 2 h, LESφ2 was the most abundant free phage detected (6.2 x 10^7^ copies μl^-1^), compared to LESφ3 (6.9 x 10^6^ copies μl^-1^) and LESφ4 (1 x 10^7^ copies μl^-1^) (Figure 1). Furthermore, the increase in LESφ2 production between 30 and 60 min exposure times was higher (3.67 fold increase) than that for LESφ3 (1.74 fold increase) and LESφ4 (2.06 fold increase). Thus while norfloxacin induction caused a significant increase in the replication of all three phages (LESφ2 - F_1, 8_ 56.97, P 0.001; LESφ3 - F_1, 8_ 14.02, P 0.006; LESφ4 - F_1, 8_ 16.88, P 0.003), only LESφ2 showed significantly greater phage production after 60 min compared to 30 min norfloxacin exposure (induction*time interaction, F_1, 8_ 20.90, P 0.002); by contrast, the duration of exposure had no effect on phage production in LESφ3 and LESφ4 (induction*time interaction, LESφ3 - F_1, 8_ 1.05, P 0.336; LESφ4 - F_1, 8_ 3.19, P 0.112). We suggest therefore that LESφ2 is either more sensitive to induction by norfloxacin or that it replicates more rapidly once induced.

### Lysogenic infection of a model PAO1 host

**Figure 2:**
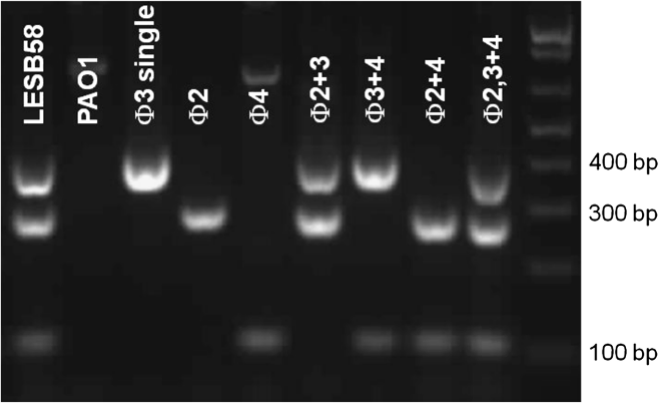
**PCR confirmation of all PAO1 LES phage lysogens.** Lysogens were isolated from turbid plaques following sequential infection of PAO1 with pure stocks of each LES phage. Lysogens were considered resistant if no plaques were observed following exposure to increasingly high titre phage suspensions (up to MOI 100). The presence of each prophage was confirmed using multiplex PCR with specific primer sets for each LES phage yielding differentially sized products: 325 bp (LESφ3); 250 bp (LESφ2); 100 bp (LES φ 4).

PAO1 LES phage lysogens (PLPLs) were created by infection of strain PAO1 with each LES phage and isolation of single colonies from turbid areas within plaques (Figure 2). Challenge of PLPLs with different LES phages, using plaque assays, revealed varying immunity profiles. Table 1 lists the efficiency of plating (eop) values of each LES phage on each PLPL lawn. Prophages 2 and 3 conferred immunity to super-infection by LESφ2 and LESφ3 respectively (eop < 1 x10^-9^). However, a few LESφ4 super-infection events were observed by detection of plaques following exposure of lysogens to 1 x 10^10^ p.f.u ml^-1^ of LESφ4 (eop = 3.33 x 10^-9^). LESφ2 was able to infect PLPLs harbouring prophages LESφ3 (eop 0.91) and LESφ4 (eop 1.09) at the same efficiency as non-lysogenic PAO1. However, lysogens harbouring the LESφ2 prophage were resistant to infection by LESφ3 (eop < 1x10^-9^) and showed considerably reduced susceptibility to LESφ4 (eop 0.017).

**Table 1:** Differential Immunity profiles of each LES phage in PAO1

**Efficiency of plating values**	**φ2**	**φ3**	**φ4**
PAO1 naive host	1.0	1.0	1.0
Single φ2 lysogen	< 1x10 ^-9^	< 1x10 ^-9^	0.017
Single φ3 lysogen	0.91	< 1x10 ^-9^	0.37
Single φ4 lysogen	1.09	0.94	3.3x10 ^-9^

Immunity profiles of each LES phage were determined by plaque assay. Phage dilution series were spotted onto non-Lysogenic PAO1 and PLPL lawns. Efficiency of plating = the ratio of plaques observed (at the appropriate phage dilution) on the most permissive host (non-lysogenic PAO1)/plaques observed on assay host (PLPL harbouring LESφ2, LESφ3 or LESφ4 prophages). If no plaques were observed when neat phage suspensions of 10^10^ p.f.u ml^-1^ were used, an eop value < 1x10^-9^ was recorded.

**Figure 3:**
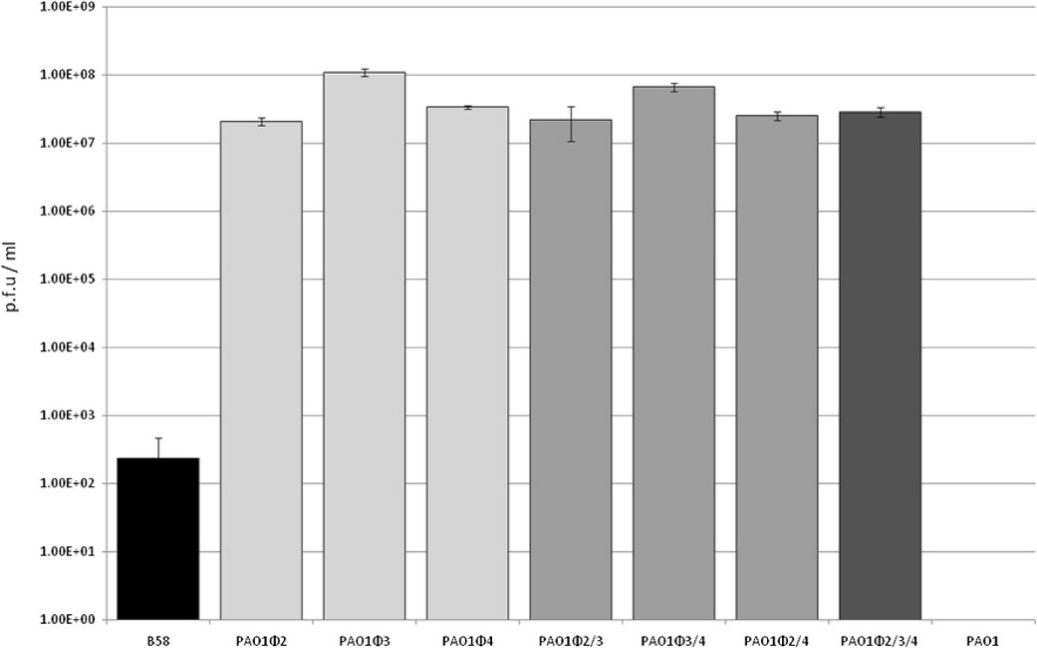
**Spontaneous lysis exhibited by LES phages in PAO1 vs LESB58.** Phage production was quantified from filtered culture supernatants of un-induced mid-exponential phase cultures using standard plaque assay. Standard deviation is shown (n = 3).

Spontaneous phage production by all seven PLPLs was higher than that associated with LESB58, by 5–6 orders of magnitude (P < 0.05) (Figure 3). These data suggest that LES prophages are less stable in PAO1, with significantly higher rates of spontaneous lytic phage production than in LESB58. Little difference was observed in the levels of spontaneous phage production between single, double and triple PAO1 lysogens.

### LES phages integrate at the same sites in different bacterial host strains

**Figure 4:**
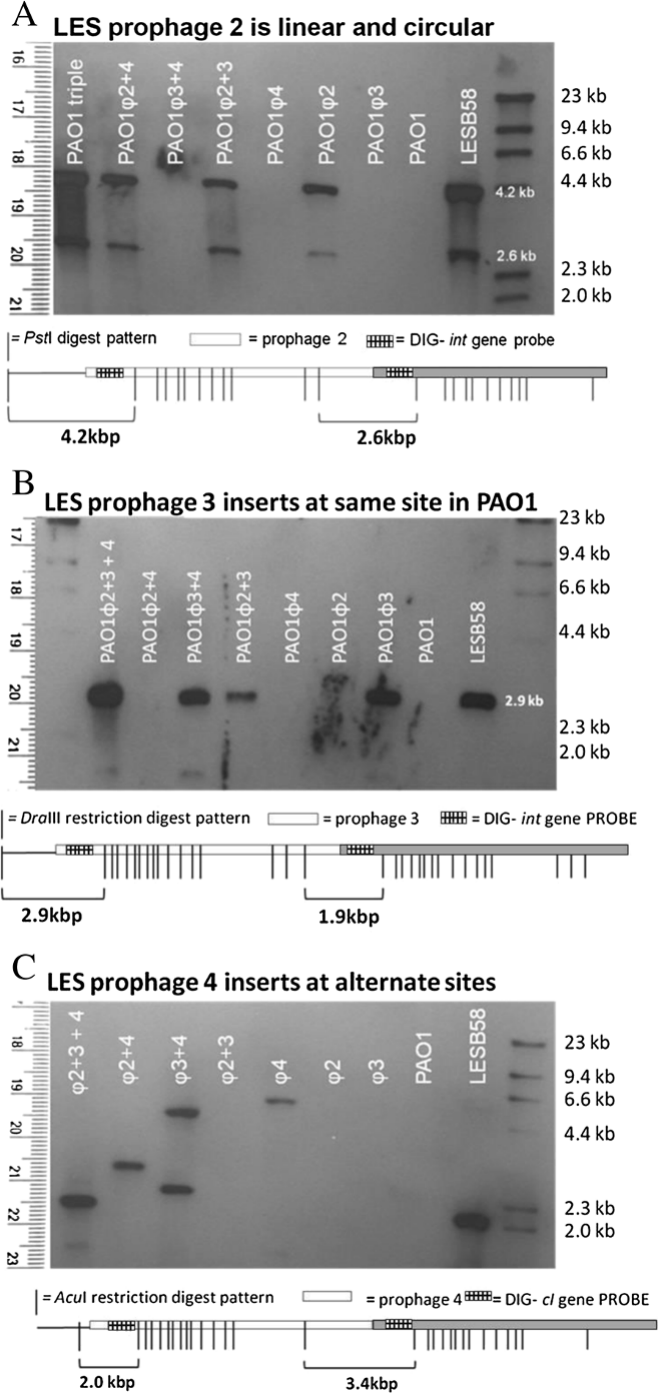
**Southern analysis of LES phage integration sites in LESB58 and PAO1.** Southern blot analysis to determine LES phage copies and integration sites in LESB58 and PLPL chromosomes: **A**) *Pst*I digested LES phage lysogens hybridised to LESφ2 integrase (int) probe; **B**) *Dra*III digested LES phage lysogens hybridised to LESφ3 integrase probe; **C**) *Acu*I digested LES phage lysogens hybridised to LESφ4 *c*I probe. A diagrammatical representation of the restriction pattern is presented below each blot. This demonstrates the expected size of fragments that would hybridise each probe in the event of single phage integration (one band) or integration of two identical prophages in tandem (two bands). For clarity, the second phage copy has been shaded in grey. The 2-band pattern would also result if any additional phage copies were present in circular form.

Southern blot analysis was used to demonstrate that lysogenic instability was not due to integration of the LES phages into unstable sites of the naive PAO1 chromosome, or from multiple integration events of the same phage (Figure 4). LESφ2 and LESφ3 integrated as single copies at identical locations in LESB58 and PAO1 chromosomes.

The LESφ2 *int* probe hybridised to an additional DNA fragment in all lysogens containing LESφ2, including LESB58. The size of the additional hybridised fragment corresponds to one of two possibilities: 1) the integration of a second LESφ2 copy in to the chromosome directly downstream of the first; 2) an extra copy of LESφ2 in circular form (Figure 4). The published LESB58 genome sequence clearly shows a single LESφ2 copy in the chromosome. Since the hybridisation pattern of the PAO1 LESφ2 lysogen matches that of LESB58, a second chromosomal copy can be ruled out. This suggests that the extra copy is circular, which may represent phage replication resulting from spontaneous activation of the lytic life cycle. Alternatively, the extra copy may indicate pseudolysogeny, in which stable circular copies are maintained.

The LESφ4 *cI* probe indicated that LESφ4 is able to integrate in several chromosomal locations in PAO1.

### LES phages infect a narrow host range in a type IV pilus-dependant manner

From a well-characterised panel of 32 clinical *P. aeruginosa* isolates, 6 were susceptible to LES phage infection. Of 25 environmental isolates, representing 17 different *Pseudomonas* species, only the *P. aeruginosa* strain was susceptible. In addition, PA14 was resistant to infection by LESφ2 and LESφ3, but susceptible to LESφ4. Plaques on PA14 appeared less turbid than those on PAO1 lawns. The host ranges of each LES phage were not identical and no correlation was found between bacterial clone-type [28] and susceptibility (data not shown). In addition, other common Gram-negative CF pathogens *Burkholderia cenocepacia* and *B. multivorans* strains were resistant to infection by all three LES phages (Table 2)*.*

**Table 2:** **Susceptibility of a panel of *Pseudomonas *** **isolates to LES phages 2, 3 and 4**

**Isolate source (#)**	**φ2**	**φ3**	**φ4**
Reference strains (2)	50% (1/2)	50% (1/2)	100% (2/2)
Keratitis patient (12)	8.3% (1/12)	0% (0/12)	33.3% (4/12)
Non-LES child (8)	12.5% (1/8)	0% (0/8)	12.5% (1/8)
Non-LES adult (6)	16.7% (1/6)	0% (0/6)	0% (0/6)
Anomalous LES (6)	0% (0/6)	0% (0/6)	0% (0/6)
Environmental (25)	0% (0/25)	4% (1/25)	0% (0/25)

Percentage of LES phage-sensitive strains as determined by plaque assay. Actual numbers tested are shown in parentheses.

A non-piliated PAO1 mutant (*pilA*^*-*^) was resistant to infection by all 3 phages, suggesting that LESφ2, 3 and 4 all require type IV pili for infection. The hyper-piliated mutant (*pilT*^*-*^) was also resistant to the LES phages, whilst an alternative hyper-piliated mutant (*pilU*^-^) remained fully susceptible.

## Discussion

### Differential induction among co-infecting prophages

Induction experiments demonstrated that LESφ2 virions were produced from LESB58 in greater numbers than the other phages. These data suggest that LESφ2 replication is more efficient than the other phages and could out number and therefore out compete the other, co-infecting LES phages during the lytic cycle. Potentially supporting this hypothesis, we detected an extra copy of this phage in the LESφ2 lysogen genome. Southern analysis suggests the presence of either a pseudo-lysogenic plasmid form [29], or a highly active replicative form of LESφ2 during spontaneous phage production.

The implications of within-host competition between co-infecting prophages has been little studied, however Refardt *et al.*[30] observed hierarchical competition between multiple prophages in *E. coli*, which suggested that the sensitivity of the lytic switch can determine dominance of one prophage over another in a polylysogen. Carriage of phages that are very prone to activation of the lytic lifecycle may represent a significant cost to their host cells, and thus could be selected against in natural populations. However, while natural isolates of LES sampled from CF patient sputa often lack one or more of the LES prophages [25], there is no evidence that LESφ2 is more or less stably maintained than LESφ3 or LESφ4.

### LES phages exhibit different immunity profiles

Each phage conferred inhibition of superinfection by the same phage, although the Mu-like phage, LESφ4 was observed to infect LESφ4 lysogens at a very low frequency. This may represent the development of rare mutations that affect immunity functions. There are several examples of such mutations in phage Mu [31]. Repressor/operator coevolution has been suggested to be the driving force for the evolution of superinfection immunity groups of lambdoid phages [32]. The same may hold true for Mu-like phages. For example, mutation of the operator region has been shown to affect binding of the repressor in Mu *vir* mutants [33].

Sequential infection of PAO1 with different LES phages revealed an interesting superinfection hierarchy. LESφ3 lysogens remained susceptible to LESφ2 and LESφ4; and LESφ4 lysogens were susceptible to LESφ2 and LESφ3. However, LESφ2 prevented infection by LESφ3 and greatly reduced susceptibility to LESφ4. Such uni-directional infection exclusion has been reported between other phages, and is commonly associated with super-infection exclusion genes such as the lambda *rex* genes [34] and *sieA*, *sieB* and *a1* in the *Salmonella* phage, P22 [35-38].

It is likely that LESφ3 and LESφ4 prophages would have been acquired before LESφ2, because the infection hierarchy suggests that prior acquisition of LESφ2 would have prevented subsequent LESφ3 and LESφ4 infection.

### LES prophages in PAO1 undergo spontaneous activation to the lytic cycle at a far higher rate than in LESB58

High levels of spontaneous induction were observed in PLPLs, suggesting that lysogeny is relatively unstable in the PAOl genetic background. We show that phage production remained high between PLPLs containing one, two or three LES prophages, suggesting that polylysogens were no more or less stable than any single lysogens. Southern analysis confirmed that LESφ2 and LESφ3 integrated into the same position in PLPLs as they did in LESB58. Therefore, the instability of PLPLs was not due to prophage integration into unstable sites. LESφ4 integrated in several alternative sites in PLPLs. The sequence of this phage shares a high level of genome synteny and homology with the transposable Mu-like phage D3112 [16], whose random integration has been demonstrated to create mutations within the host chromosome. LESφ4 may play a similar role in LES genome evolution.

### The LES phages exhibit a narrow host-range

Our investigation of the LES phage host range revealed narrow, overlapping host specificity. No association between bacterial clone-type and phage susceptibility was observed, although testing more strains may have identified a pattern. Despite the high proportion of resistant clinical isolates, our data show that LES phages are capable of infecting some *P. aeruginosa* strains isolated from keratitis patients and non-LES infected CF patients. LES phages have been detected in CF patients’ sputa, and may therefore allow mobilisation of genes between *P. aeruginosa* strains [25,26]. By contrast, LES phages may allow LES to displace other *P. aeruginosa* strains during superinfection in the CF lung [11] by lysing susceptible resident strains [39].

### LES phage infection is Type IV pilus-dependent

We demonstrate that LES phage infection is dependent on the type IV pilus, which is required by *P. aeruginosa* for adhesion, biofilm formation and twitching motility [40-42]. This important surface structure is commonly used as a receptor by diverse *Pseudomonas* phages [43]. Both non-piliated (*pilA*^*-*^) and hyper-piliated (*pilT*^*-*^) PAO1 mutants were resistant to infection by all three LES phages. However, a different hyper-piliated mutant (*pilU*^*-*^) remained susceptible. These findings mirror other pilin-dependent *P. aeruginosa* phage studies [43-45]. Hyper-piliated mutants are incapable of twitching motility due to abrogated pili retraction. These data suggest that retraction is involved in the infection process by LESφ2 LESφ3 and LESφ4.

Despite infecting via an important and common surface structure, all three LES phages exhibited narrow host ranges and each showed strain specificities. For example, LESφ4 was able to infect PA14 and several keratitis isolates that were resistant to infection by the other LES phages. It is likely that many clinical strains of *P. aeruginosa* harbour prophages that may belong to the same immunity group and therefore exclude super-infection by one or more of the LES phages [20]. Alternatively, resistance could be achieved by loss or modification of the type IV pili receptor [44,45].

## Conclusion

In summary, we demonstrate that the LES phages exhibit differential sensitivities to induction, narrow host ranges and divergent infection behaviour in the model host PAO1 compared with the native LESB58 host background. Extensive genotypic and phenotypic variation has been observed in clinical LES populations [46], including changes in the number of resident LES prophages [25]. These phages may, therefore, be important contributors to diversity of the LES populations.

## Methods

### Bacterial strains and growth conditions

All bacterial strains used in this study and their sources are listed in Table 3. LES phages were induced from the sequenced CF *P. aeruginosa* isolate, LESB58 [16]. Strain PAO1 was susceptible to infection by all three LES phages and was therefore used as a model host to purify and study the characteristics of each phage. Successive infection of PAO1 with purified LES phages yielded single, double and triple PAO1 LES Phage Lysogens (PLPLs) each harbouring single copies of one, two or three LES phages simultaneously. All lysogens were confirmed by PCR amplification of specific prophage sequences and Southern blot analysis. Non-piliated (*pilA*^*-*^) or hyperpiliated (*pilT*^*-*^ and *pilU*^*-*^) PAO1 mutants [47] were used to determine whether LES phages infect via the type IV pili. All bacterial strains and phages were grown and propagated in standard lysogeny broth (LB) at 37°C (clinical isolates) or 23°C (environmental isolates). Phage suspensions were stored in LB at 4°C. 

**Table 3:** Bacterial strains and sources

**Strain (**^**1**^**Clone type)**		**Reference/source**
**Laboratory *P. aeruginosa *** **strains:**		
PAO1(W)		[2]
PAO1 *pilA*-; PAO1 *pilU-*; PAO1 *pilT-*^*2*^		[47]
PA14(A)		[48]
**Clinical LES isolates:**		
LESB 58 (T) - Sequenced isolate		[16]
LES 431 (T) - Lacks LES prophage 2		[49]
**Anomalous LES isolates**^3^**:**		
O69574 (T); 0521 (T); 43513 (T); 079444 (T); 0342 (T).		[50]
***P. aeruginosa *** **isolates from keratitis patients**^4^**:**		
39015 (B); 39115 (A); 39103 (A2); 39145 (A3); 39053 (A5); 39135 (C); 39016 (D); 39421 (F); 39061 (I); 39284 (L); 39376 (U); 39129 (V).		[51]
***P. aeruginosa *** **isolates from non-LES infected CF patients:**		
**CHILDREN:** AH23 (B); AH4 (A); AH19 (A3); AH14 (C); AH1 (D); AH6 (L); AH9 (U); AH7 (A4);		AHCH^5^
**ADULTS:** NL28 (A); NL20 (C); NL25 (F); NL16 (U); NL21 (A4); NL14 (A7).		RLUH^6^
**Environmental *Pseudomonas spp*:**	Strain	
*P. aeruginosa*	159	RJ^7^
*P. fluorescens*	WC5365; F113; ATCC 17400; pf5; pf01.	
*P. syringae*	*‘tomato’* DC300; B728a	
*P. syringae pv. Coriandricola*	Ccola	
*P. syringae pv. maculocola*	M4	
*P. syringae pv. antirrhini*	152E	
*P. putida*	KT2440; *Paw*340	
*P. cichori*	907	
*P. avellanae*	48	
*P. phaseiolicola*	1448A	
*P. entomophila*	L48	
*P. marginalis*	247	
*P. corrugata*	2445	
*P. tolaasii*	2192 T	
*P. glycinea*	49a/90	
*P. lachrymans*	789	
*P. agarici*	2472	
*P. viridiflava*	2848	
*B. cenocepacia*	K56-2; J2315.	[52]
*B. multivorans*	F-A1-1; LMG 13010.	

^1^Clones typed using the Clondiag tube array system [51]; ^2^ PAO1 *pil* mutants acquired from Angus Buckling, University of Exeter. ^3^Isolates classified as anomalous following negative diagnostic PCR result for one of two specific target sequences, but identified as LES using the tube array system. These isolates were also missing one or more LES prophage. ^4^ Strains isolated from Keratitis patients from several hospitals across the UK. ^5^ AHCH: Isolates collected from child CF patients attending the Alder-Hey Children’s Hospital, Liverpool. ^6^ RLUH: Isolates collected from adult CF patients attending the Royal Liverpool University Hospital. ^7^ RJ ^-^Environmental isolates of several *Pseudomonas* species donated by R Jackson, University of Reading.

### Bacteriophage induction

*P. aeruginosa* LESB58 was grown to mid-exponential phase (OD_600_ 0.5) and LES phages were induced into the lytic cycle by exposure to the minimum inhibitory concentration of norfloxacin (50 μg ml^-1^) for 1 h [24]. Induced cultures were sub-cultured (1:10) into fresh LB to enable recovery for 2 h before filtration (0.2 μm Millipore). Active phage particles in the induced supernatants were enumerated by standard plaque assay using PAO1 host cells.

### Bacteriophage assays

LES phages were isolated from induced LESB58 cultures using plaque assays with PAO1 host cultures as described previously [24]. Phages were purified by picking individual plaques that were suspended in LB (1 ml), filter sterilized (0.2 μm Millipore) and used in a second plaque assay with PAO1. This process was repeated twice to ensure purity. Phage purity was confirmed using PCR assays. Amplification of phage stocks was achieved by modifying previous methods [53]. Briefly, mid-exponential phase PAO1 cultures (100 ml) were infected with purified LES phage (MOI = 0.1), at 37°C for 2 h. Lysed cultures were filter-sterilized.

### Electron microscopy

Phage suspensions (1x10^9^ – 1x10^10^ p.f.u. ml^-1^) were concentrated by centrifugation, negatively stained with 2% (w/v) uranyl acetate [54], and examined by transmission electron microscopy (magnification x 200,000).

### Multiplex PCR to confirm pure phage stocks and lysogens

Three primer sets, LESnest1 F/R, Clust6nest F/R and 4tot1 F/R (Table 4), for the detection of LES phages 2, 3 and 4 respectively, were combined in a multiplex PCR assay for confirmation of each pure phage stock and each PLPL. Colony or filtered phage suspensions were used as templates in each reaction as described previously [25]. 

**Table 4:** Primer sequences

**Primer**	**Sequence (5′-3′)**	**Amplicon (bp)**	**Cycling conditions**	**Reference**
Multiplex PCR:				
LES1nestF	tttggtgatgatcggcttagc	289	95°C, 4 min then 30 cycles: 95°C, 30 s; 58°C, 30 s; 72°C, 30 s; final extension step, 72°C, 7 min;	[25]
LES1nestR	tgtggaagcgatcagtct			
Clust6nestF	ggatcgacgtggcataatctg	410		[25]
Clust6nestR	acgattctccggcatgcagcg			
4tot1F	gctcatgagtggctgacaac	105		This study
4tot1R	tcttgggcagagaaccattc			
Q-PCR:				
2pro3F	caagccctgtctggattttc	102	95°C, 10 min; then 40 cycles: 95°C, 10 s; 60°C, 15 s; 72°C s.	This study
2pro3R	gagacaggttgggagggagt			
3tot1F	cgcaggtaccaccagacttt	122		This study
3tot1R	catgtccagcaggttcaaaa			
3pro3F	gcggatgttctcaaacgaat	134		This study
3pro3R	cgggagaagcaatgacctac			
4tot1F	gctcatgagtggctgacaac	105		This study
4tot1R	tcttgggcagagaaccattc			
4pro3F	tcgtgctgtgctgatctttt	172		This study
4pro3R	agcagtgccagttgatgttg			
Preparation of DIG-labeled probes:				
φ2*int*DIGF	tgcctatctaacggggttca	1097	95°C, 4 min. 30 cycles: 95°C, 30 s; 55°C, 30 s; 72°C, 1 min s; final extension step, 72°C, 7 min	This study
φ2*int*DIGR	gaagcaaccgagaagtggag			
φ3*int*DIGF	ggatcatgtagcgggaaaga	874		This study
φ3*int*DIGR	agaacctggcgaaagtctga			
φ4*cI*DIGF	atcgttaattggcacggaat	893		This study
φ4*cI*DIGR	acagcaacggatttccactc			

*tot* = to quantify total phage copies; *pro* = to quantify total phage copies.

### Quantifying production of each LES phage from LESB58

Replication of each LES phage in response to induction of the lytic cycle was compared using Q-PCR to distinguish and enumerate each specific phage type. LESB58 induction experiments were performed on three separate occasions in the presence and absence of norfloxacin for 30 and 60 min exposure times before the 2 h recovery step. DNA was prepared from each replicate using the Bacterial and Virus DNA extraction kit (QIAGEN) and the automated QIAsymphony machine (QIAGEN; pathogen complex 200 protocol). Q-PCR was performed using six specific primer sets to differentiate between prophage and total copies of each phage.

### Real-time Q-PCR

Q-PCR was used to quantify LES phages by comparing the number of specific amplicon copies in extracted DNA from induction experiments to a concentration gradient of known standards. Primer sets with the prefixes, “tot” (total) and “pro” (prophage) were designed to amplify unique regions within, and flanking, each LES phage genome (Figure 1D). All primer sequences and amplification details are listed in Table 4. Amplicon copy number μl^-1^ was calculated using the formula [(6.023 x 10^23^ x [DNA] g/ml)/(molecular weight of product)]/1,000 [55]. Molecular weight was calculated as number of base pairs x 6.58 x 10^2^ g. A 10-fold dilution series of each DNA standard was prepared for quantification of phage numbers in each sample.

Q-PCR reactions (25 ul) contained 1 uM each primer pair and 1X Rotorgene-SYBR green supermix (QIAGEN). Phage numbers were quantified from DNA samples (1 μl) in triplicate using a Rotorgene cycler (QIAGEN). Q-PCR data were analyzed using Rotorgene Q series software 1.7 (QIAGEN). Total phage and prophage numbers from each sample were quantified in separate reactions using “tot” and “pro” primer sets for each phage and comparing fluorescent signals to those from standard concentration gradients. The level of free phage in a given sample was calculated by subtracting prophage numbers from total phage numbers.

### Statistical analysis

Specific phage sequences were quantified in triplicate from each of 3 experimental replicates using Q-PCR, and technical replicates were averaged prior to analyses. Differences in phage numbers, with and without norfloxacin and between time-points were analysed using separate ANOVAs for each phage, fitting induction (2 levels), time (2 levels) and their interaction as fixed factors.

### Isolation of PAO1 lysogens

PAO1 LES phage lysogens (PLPLs) were isolated from turbid islands in the centre of well-separated plaques using a sterile toothpick and streaked on to Columbia agar (Oxoid) to obtain single colonies. Individual lysogen colonies were analysed by multiplex PCR assays to confirm the presence of LES prophages.

### Immunity assays

Lawns of PAO1 and each PLPL were created by adding mid-exponential phase (OD_600_ 0.5) cultures (100 ul) to molten 0.4% (v/v) agar and pouring onto Columbia agar plates to set. A 10-fold dilution series of each purified phage suspension (10^10^ – 10^3^ p.f.u ml^-1^) was spotted (20 ul) onto lawns of each host. Countable plaques were observed at varying dilutions depending on the phage-host combination. The efficiency of plating (eop) value was calculated as the ratio of assay titre/most permissive titre. The most permissive titre was obtained on non-lysogenic PAO1.

### Southern blot analysis

Southern analysis was performed as previously described [56]. Specific probes were prepared using the digoxigenin (DIG) PCR labelling kit (Roche). DIG-labelled probes were designed to hybridize targets within either the LESφ2 *int* gene, the LESφ3 *int* gene, or the LESφ4 *c*I gene using primers: φ2*int*DIG F/R; φ3*int*DIG F/R and φ4*cI*DIG F/R (Table 4). Bacterial genomic DNA was extracted using a Wizard Genomic DNA extraction kit (Promega) and digested using *Pst*I*, Acu*I or *Dra*III (NEB) according to the manufacturer’s instructions. Probes were hybridised to digested genomic DNA as described previously [53]. Hybridized probe was detected using alkaline phosphatase-conjugated anti-DIG antibody (1:10,000) and CPD*star* substrate (1:100) (Roche) according to the manufacturer’s instructions.

## Competing interests

The authors declare that they have no competing interests.

## Authors’ contributions

CJ designed the study; carried out the purification and characterisation of the LES phages and rates of induction and drafted the manuscript. JL carried out initial induction of the phages from the native host. HK and CJ carried out the host range study. AH clone-typed each clinical *P. aeruginosa* isolate. JC prepared samples for electron microscopy of LESφ2 and LESφ3. MB and CW jointly conceived of the study and participated in its design and coordination and helped to draft the manuscript. All authors read and approved the final manuscript.
